# Relationship between polyclonal immunoglobulin therapy and colonization by *Candida *spp.

**DOI:** 10.1186/cc10655

**Published:** 2012-03-20

**Authors:** G Serafini, I Cavazzuti, C Venturelli, M Girardis

**Affiliations:** 1 University Hospital, Modena, Italy

## Introduction

Low IgA levels in blood serum and in saliva have been associated with an increased risk for Candida colonization and infection [1,2]. In this retrospective cohort study, we aimed to evaluate the effects of an intravenous immunoglobulin preparation containing polyclonal IgG, IgM and IgA (IgGAM) on the prevention of *Candida *spp. colonization in patients with septic shock.

## Methods

In this study we analyzed 69 patients with septic shock and without *Candida *spp. colonization before shock appearance admitted to the ICU of a university hospital from January 2008 to November 2011. All of the patients were treated in according to the Surviving Sepsis Campaign guidelines. In addition to standard therapy, 44 (64%) patients received IgGAM therapy (Pentaglobin^® ^38 g/l IgG, 6 g/l IgM, and 6 g/l IgA) within 24 hours from the diagnosis of septic shock at the dose of 250 mg/kg/day for 3 days. The colonization by *Candida *spp. was evaluated by analyzing the results of the microbiological surveillance cultures (two times per week) of pharyngeal swab, tracheal aspirate, urine and surgical drains between 48 hours and 21 days after the diagnosis of septic shock.

## Results

In the IgGAM group, 11 patients (25%) developed *Candida *spp. colonization compared to nine patients (36%) of the control group. The Candida colonization index was similar in the two groups: 0.42 ± 0.16 in the IgGAM group and 0.45 in the control group.

## Conclusion

The above data show a slight reduction of *Candida *spp. colonization in septic shock patients treated with IgGAM therapy. Further studies are needed to confirm this finding.

## References

[B1] Gonçalves e Silva CR Melo KE Leão MV Ruis R Jorge AO Relationship between Candida in vaginal and oral mucosae and salivary IgA Rev Bras Ginecol Obstet 2008 30 300 305 10.1590/S0100-72032008000600006 19142508

[B2] Bai XD Liu XH Tong QY Intestinal colonization with *Candida albicans *and mucosal immunity World J Gastroenterol 2004 10 2124 2126 15237449 10.3748/wjg.v10.i14.2124 PMC4572348

